# Plant Virology Delivers Diverse Toolsets for Biotechnology

**DOI:** 10.3390/v12111338

**Published:** 2020-11-23

**Authors:** Mo Wang, Shilei Gao, Wenzhi Zeng, Yongqing Yang, Junfei Ma, Ying Wang

**Affiliations:** 1 Fujian University Key Laboratory for Plant-Microbe Interaction, Fujian Agriculture and Forestry University, Fuzhou 350002, China; gaoshilei1996@163.com; 2 Key Laboratory of Ministry of Education for Genetics, Breeding and Multiple Utilization of Crops, College of Agriculture, Fujian Agriculture and Forestry University, Fuzhou 350002, China; fafuzwz@163.com; 3 Root Biology Center, Fujian Agriculture and Forestry University, Fuzhou 350002, China; yyq287346@163.com; 4 Department of Biological Sciences, Mississippi State University, Starkville, MS 39759, USA; jm5026@msstate.edu

**Keywords:** plant virus, viroid, viral vector, virus-induced gene silencing (VIGS), CRISPR/Cas9, genome editing, carotenoid biosynthesis, vaccine, circular RNA

## Abstract

Over a hundred years of research on plant viruses has led to a detailed understanding of viral replication, movement, and host–virus interactions. The functions of vast viral genes have also been annotated. With an increased understanding of plant viruses and plant–virus interactions, various viruses have been developed as vectors to modulate gene expressions for functional studies as well as for fulfilling the needs in biotechnology. These approaches are invaluable not only for molecular breeding and functional genomics studies related to pivotal agronomic traits, but also for the production of vaccines and health-promoting carotenoids. This review summarizes the latest progress in these forefronts as well as the available viral vectors for economically important crops and beyond.

## 1. Introduction

In 1898, the discovery of tobacco mosaic virus (TMV) as the causative agent for the tobacco mosaic disease marked the birth of virology and expanded the knowledge of life domains [1]. In 1939, TMV was observed under an electron microscope, providing the first image of a virion in history [2,3,4]. In 1957, Fraenkel-Conrat and coworkers elegantly demonstrated that RNA, akin to DNA, can serve as genetic material, using TMV infecting tobacco plants as a model system [5]. In 1971, the discovery of potato spindle tuber viroid as the causative agent for potato spindle tuber disease further expanded the knowledge of pathogens and established the minimal inheritable genome in biology [6]. Beyond those milestone discoveries, studies on plant viruses and viroids have significantly contributed to the development of numerous recent research forefronts, including, but not limited to, epigenetics [7] and RNA silencing [8,9].

**Figure 1:**
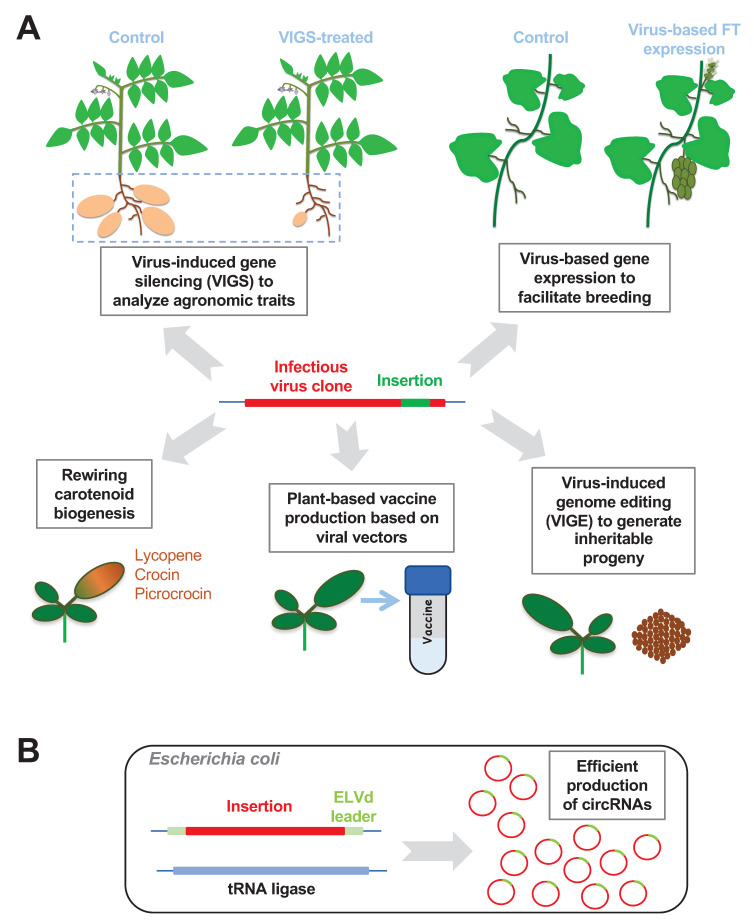
Plant virus/viroid-based technology. (**A**) Application of plant virus vectors in agriculture and production of carotenoids and vaccines. Virus-induced gene silencing (VIGS) has been used to characterize genes controlling important crop traits, exemplified by tuber formation (highlighted by blue dashed lines) in potato plants. Virus-based gene expression of FT (Flowering Locus T) can induce early flowering in grapevines, shortening the time for molecular breeding. VIGE in plants can shorten the time in generating stable transgenic progeny. Viral-based expression platform can be used for the production of vaccines and health-promoting carotenoids. (**B**) Viroid-based platform for circular RNA production. ELVd, eggplant latent viroid. circRNA, circular RNA.

With mounting knowledge about plant viral gene functions and plant–virus interactions, many plant viruses have been successfully developed as biotechnology tools (Figure 1A). For instance, plant viruses have been harnessed as RNA silencing vectors for functional studies on genes underlying desired crop traits [10,11,12,13,14]. Recently, numerous plant viral vectors have been developed for CRISPR/Cas9-based genome editing of model and crop plants [15]. Furthermore, plant viral vectors have been developed to express endogenous and foreign polypeptides in controlling agronomic traits or producing vaccines and valuable carotenoids benefiting human beings [16,17,18]. Compared with traditional transgenic approaches, plant viral vectors can markedly reduce the time and cost in modulating gene expression, thereby having great potential in agricultural and biomedical applications [16].

In this review, we summarize the plant viral vectors designed for virus-induced gene silencing (VIGS), genome editing, and exogenous protein expression in major crops. In addition, we introduce the current status and progress of the plant virus-based production of vaccines and health-promoting carotenoids. Furthermore, we outline the viroid-based production of circular RNAs for research applications.

## 2. A brief Overview of Plant-Virus Interactions

Plant diseases caused by viruses are economically important, as they seriously affect the quality and yield of cereals, vegetables, and fruits. All the food, feed, fiber, ornamental, and industrial crops are threatened by at least one virus, and the great losses caused by plant virus diseases are second only to that by fungal diseases [19]. Plant viruses are obligate parasites, which lack protein-synthesizing and energy-producing apparatuses, and extensively depend on the host machinery for their replication [20]. Virus particles, also called virions, consist of two basic components: the nucleic acid genome and a protective protein coat. In general, viral genomes encode the minimal set of genes critical for infection, such as polymerases, coat proteins, movement proteins, etc. Interestingly, a peculiar group of noncoding RNAs, termed viroids, can cause plant disease without encoding any protein or being encapsidated.

The infection cycle of plant viruses starts from their penetration into host cells. Because plant viruses and viroids by themselves cannot breach the plant physical barriers (i.e., cuticle and cell wall), they are only able to enter the host cells passively through opportunistic mechanical wounds or with the aid of insect vectors (e.g., aphids or whiteflies) [20,21,22]. After they successfully enter cells, the following infection process can be artificially divided into four major steps [23]. The first step is the disassembly of viral particles, which is the partial or complete removal of coat proteins to release viral genomes into host cells [24]. The second step is the host cell-dependent replication of viral genomes and the translation of viral proteins [25,26,27]. In this process, plant viruses must recruit and utilize the host’s translation apparatus [28] as well as the host’s energy resources [29]. Some plant viruses, particularly single-stranded DNA (ssDNA) geminiviruses, rely on host polymerases for genome replication [30,31]. In the third step, viral genome encapsidation occurs to form new virions [32,33]. The last step is the cell-to-cell movement and long-distance trafficking to successfully colonize an entire plant [34,35,36,37,38]. 

In plants, RNA silencing plays a major role in defending viral infections [39], in addition to innate immunity [40]. It is generally accepted that viral replication intermediates form double-stranded RNAs (dsRNA) to activate plant RNA silencing. Viral dsRNAs are processed to viral short interfering RNAs (vsiRNAs) by plant dsRNA-specific RNases, Dicer-like enzymes (DCLs). VsiRNAs are then efficiently loaded into Argonaute proteins (AGOs) to form the antiviral RNA-induced silencing complexes (RISCs), which subsequently target viral RNAs via slicing or translational arrest [39,41]. Successful viral infection relies on the activity of virus-encoded viral suppressors of RNA silencing (VSRs) [42]. Despite the fact that viroids do not encode any proteins nor possess VSR activity, they can establish successful infections, which is likely attributable to their highly structured RNA genomes and differential subcellular localization of sense and antisense viroid RNAs [43].

## 3. Engineering VIGS Vectors

Taking advantage of the robust production of vsiRNAs, multiple infectious clones of plant viruses have been engineered to include fragments of endogenous genes for RNA silencing, termed VIGS [10,11,12,13,14]. As listed in Table 1, there are multiple strategies for generating viral vectors. The engineered viruses should retain infectivity and incite mild symptoms. In line with this consideration, non-structural genes or pathogenicity determinant factors are often replaced with cloning sites. For instance, the tobacco rattle virus (TRV)-based VIGS vector was engineered by removing two non-structural genes in RNA2 [44], whereas the tomato yellow leaf curl China virus (ToLCCNV)-based VIGS vector was engineered by removing the pathogenicity determinant factor βC1 in DNA β [45]. Coat/capsid protein genes are popular choices for modifications as well, by either completely being replaced by a multiple cloning site [46] or being partially truncated for insertion of cloning sites [47]. These modifications generally have minimal impacts on viral infectivity. For viruses expressing subgenomic RNAs, it is common to duplicate the subgenomic RNA promoters to flank a multiple cloning site [48,49,50]. This strategy can lead to the production of new subgenomic viral RNAs that have less impact on viral infectivity. Notably, the insertions can be designed to form a hairpin structure composed of inverted duplication of sense and antisense sequences of target genes to enhance silencing effects, if a duplicated subgenomic RNA promoter is harnessed [49]. Some viruses belonging to the same family may be engineered via the same or similar strategy. For instance, it is common to duplicate the protease cleavage site in the polyprotein to flank an inserted multiple cloning site for viruses of the family *Secoviroidae* [51,52,53]. 

The engineered VIGS constructs are commonly delivered to plants via agro-infiltration or mechanically inoculation of in vitro transcribed RNA. Infection of engineered viruses results in abundant small RNAs from the inserted fragments that suppress the expression of host genes being targeted, based on the sequence homology. Because of its high efficiency and ease of handling, VIGS has been extensively used in plant functional studies, particularly in the species where the stable transformants are not easy to obtain [10,11,12]. 

## 4. VIGS for Rapid and Transient Gene Silencing in Plants/Crops

The host range of the parental wild-type viruses determines the plant species where these VIGS vectors can be used. VIGS vectors developed in the early days are mainly derived from TMV, potato virus X (PVX), and TRV, which are initially utilized in silencing genes in *Nicotiana benthamiana* and tomato (*Solanum lycopersicum*). Over the last two decades, more than 40 viruses have been developed as VIGS vectors for dicot and monocot plant species. Among those, more than 20 have been used for economically important crops, as summarized in Table 2. These available tools markedly shorten the time for functional assays in identifying genes related to desired traits in economically important crops, greatly facilitating the breeding efforts. Readers are referred to the collection of extensive reviews for more details [11,14,65].

Although VIGS provides a convenient approach to manipulate gene expressions in plants, it is important to note that these vectors represent infectious viruses. Despite the fact that most of them do not cause drastic phenotypic alterations, they may still affect gene expression in hosts. A recent study showed that TRV-based viral VIGS vectors alone can trigger changes in the alternative splicing of host genes, slicing activity of a plant microRNA (miR167), as well as the expression of plant mRNAs, phased secondary siRNAs, and long noncoding RNAs [66]. Thus, proper controls and considerable cautions need to be taken into account when analyzing experimental data based on viral silencing vectors. Furthermore, off-target effects can occasionally render the data annotation complicated [67].

## 5. Plant Virus-Based Tools for Plant Genome Editing

CRISPR/Cas nucleases-based genome editing technologies have provided unprecedented power in plant breeding to accelerate the manipulation of desired crop traits. The single-guide RNA (sgRNA) directs the Cas nucleases to target the genome regions introducing designed mutations. The traditional experimental process requires extensive tissue culture handlings and prolonged selection to remove the transgenic copy of the CRISPR/Cas cassette. The tissue culture handlings for many major crops are technically challenging and demanding. More importantly, the limited expression of sgRNA expression in tissue cultures leads to low efficiency in genome editing [15].

Geminiviruses, a group of ssDNA viruses that replicate in the nucleus, were soon exploited to express sgRNAs to boost the efficiency after the CRISPR/Cas nucleases-based technology became available [84]. This type of virus-based strategy in gene editing is termed virus-induced genome editing (VIGE). Interestingly, TRV, an RNA virus that replicates in the cytoplasm, was also capable of delivering sgRNA for genome editing in the nucleus [85]. Since 2014, various viral vectors have been developed for genome editing of important crops, such as potato, tomato, wheat, rice, maize, etc. (Table 3). Because the Cas nuclease genes are too large (~4.2 Kb) to insert into many viral vectors, most of these efforts rely on introducing the Cas nucleases into plants via traditional transgenic approaches or expressing Cas nucleases in a different vector. There are several approaches to incorporate sgRNAs into viral vectors. A popular choice is to place the sgRNA scaffold under the control of plant U6 gene promoter [84,86,87,88,89,90]. However, the U6 promoter occasionally results in weak expression of sgRNAs [91]. Recently, several reports used tRNAs to flank the sgRNA scaffold [92,93], and the tRNAs were subsequently removed by the activity of endogenous tRNA processing enzymes (RNase P and RNase Z) [94]. Notably, tRNA-flanking may not be needed based on the experimental practice when using some viral vectors [93,95,96].

Most viral vectors, by and large, only perform gene editing in local infection sites or protoplasts. Therefore, they do not lead to inheritable traits in the progeny. To circumvent this shortcoming, a recent study used Agrobacteria harboring the foxtail mosaic virus constructs to inoculate *N. benthamiana* seeds with the seed coat manually cracked, which resulted in the progeny inheriting the edits [86]. One critical factor to effectively generate inheritable genome-edited plants relies on the delivery of sgRNAs to germlines. A recent attempt employed a portion of the Flowering Locus T (FT) mRNA to promote the entry of sgRNAs to reproductive organs, thereby increasing the efficiency of the inheritable genome edits [97]. Although this approach indeed increased the frequency of the inheritable genome edits, the mechanism remains to be further elucidated, as the protein product of the FT gene, but not its transcripts, are the mobile signal [98,99,100,101,102,103]. Very recently, sonchus yellow net rhabdovirus (SYNV) was employed as a VIGE vector for tobacco plants [92]. This system, by far, provides the easiest and most robust DNA-free approach in generating plants bearing inheritable genome edits through simple leaf inoculations. The analysis also showed that the off-target effects are minimal through this approach. Moreover, the viral vector is stable through mechanical transmission/passages and can be easily eliminated after seed set, therefore preventing potential deleterious effects caused by the vectors [92]. Despite the fact that the host range restriction of SYNV limits its application in various crop species, this progress already demonstrates the great promise of VIGE in application.

Recent studies demonstrated that the ectopic expression of CRISPR/Cas nucleases in plants is subject to negative regulation by the RNA silencing machinery, hindering genome editing efficacy [107,108]. Notably, genome editing efficiency can be improved by including an artificial microRNA cassette in vectors to down-regulate the expression of key players in post-transcriptional gene silencing (e.g., RDR6 and AGO1) [107,108]. Similarly, RNA silencing suppressors cloned from plant viruses can also increase genome editing efficacy of either VIGE [86,104] or the transgene-based approach [108].

## 6. Plant Virus-Based Gene Expression Vectors 

In addition to serving as the VIGS and VIGE vectors, most viral vectors listed in Table 1 and Table 3 can be exploited for expressing heterogeneous proteins in plants. In the early days, plant viral vectors were based on the “full-virus” vector strategy to express genes-of-interest fused with a viral gene (e.g., coat protein gene in TMV) [109]. These viral vectors retain the full capacity of replication, assembly of virions, cell-to-cell movements, and resistance to host gene silencing [109]. The non-cell-autonomous nature of viruses can turn almost the entire plant into a factory for foreign protein synthesis. The expression level of foreign peptides can reach up to 10% of total soluble protein [109]. However, the insertion size limitation restrains the application of many viral vectors. Proteins larger than 30 kDa are difficult to express using the “full-virus” vector strategy [109]. To circumvent this shortcoming, it is common to replace certain viral non-structure genes or pathogenicity determinant factors with a multiple cloning site for large insertions as aforementioned. Another strategy employs a recombination system to deconstruct viral genes for generating a set of expression vectors [110]. In this system, the viral sequence is engineered to replace the coat protein gene with a LoxP recombination site. The gene to be expressed is placed in a separate vector downstream of another LoxP site. Both viral vectors and the plasmid harboring the expressing gene are mixed and co-infiltrated with a third vector to express the Cre recombinase [111]. This system further increases protein yield up to nearly 50% of total soluble proteins and facilitates the expression of larger foreign genes. However, since the viral elements are kept to a minimum, the deconstructed viral vectors can only be expressed in local leaves [111].

Plant virus-based protein expression vectors have been widely used in basic sciences to understand plant gene functions [11,112]. Moreover, these vectors have great application in altering agronomic traits as well. For instance, apple latent spherical virus was engineered to express the FT gene, which successfully promotes the early flowering of grapevine [113] and strawberry [79]. Similarly, citrus leaf blotch virus was used to express FT and prompt the early flowering of citrus plants [114]. This approach significantly accelerates the breeding process. More importantly, the capacity of plant viral vectors to promptly alter agronomic traits opens up many possibilities for precision agriculture.

## 7. Rewiring Plant Metabolic Pathways for the Production of Health-Promoting Carotenoids

Many plant secondary metabolites are useful nutrients or health-promoting molecules. However, those beneficial metabolites are often accumulated at low levels. For instance, crocins, which are carotenoid derivatives serving as a valuable spice and potent pain reliever, are mainly accumulated in the stigma of *Crocus sativus* L. flowers or, to a lesser extent, in gardenia fruits [115]. Due to the labor-intense procedures in collecting flowers, it is costly to produce crocins and the related molecules, such as picrocrocin. Using a tobacco etch virus (TEV)-based vector, specific cleavage dioxygenase enzymes (CCDs) in crocins biogenesis cloned from *C. sativus* or *Buddleja davidii* were expressed in *N. benthamiana* plants, resulting in a significant accumulation of crocins and picrocrocin [115]. The yield was further improved when the CCD from *C. sativus* was co-expressed with other carotenogenic enzymes (e.g., phytoene synthase from *Pantoea ananatis* and β-carotene hydroxylase 2 from saffron). This unique TEV vector removes the essential viral gene NIb (nuclear inclusion b) to gain the capacity for large insertions [116]. Consequently, this viral vector can only infect transgenic plants expressing NIb, which prevents the viral vector from entering the environment. Using the same TEV system, a soil bacterial gene cassette consisting of GGPP synthase, phytoene synthase, and phytoene desaturase was expressed in *N. benthamiana*, resulting in significantly increased production of lycopene in the cytoplasm [117]. Lycopene is a major carotenoid in human blood protecting against oxidative damage. Given the difficulties in rewiring carotenoid metabolism using traditional transgene approaches [118,119,120], viral vector-based production provides a plausible solution for engineering plant metabolic pathways with low cost and excellent performance.

## 8. Plant Virus-Based Production of Vaccines

Plant viral vectors have also been successfully harnessed in producing vaccines against devastating pathogens infecting human beings and livestock [18,121]. The surface antigen of human hepatitis B virus expressed in transgenic tobacco can form virus-like particles (VLPs) in plants [122], and those VLPs are capable of inducing potent B-cell and T-cell immune responses in mice [123,124]. Encouraged by this finding, viral vectors have been developed and employed for the rapid and robust production of various vaccines [18,121]. The target antigens can be expressed using plant viral vectors as either epitope presentation by displaying the recombinant epitope-coat protein on the surface of the chimeric virions or the polypeptides alone [18]. In 1995, the antigenic peptide of canine parvovirus VP2 protein was successfully expressed in plants, which can elicit high levels of neutralizing antibodies in mice and rabbits [125]. Since then, over a dozen antigenic peptides have been successfully expressed in plants against various pathogens, such as influenza virus, West Nile virus, hepatitis A and B viruses, human immunodeficiency virus, etc. [18,126]. A few vaccines or pharmaceutical proteins synthesized in plants using plant viral vector systems, such as the Newcastle virus subunit vaccine, have been approved for markets [126]. During the current COVID-19 pandemic, plant viral vector-based vaccine production may provide a convenient platform for production when some COVID-19 vaccine candidates prove to be safe and effective [127]. 

Notably, vectors based on plant RNA viruses are popular choices for expressing antigens. TMV [128], cowpea mosaic virus [129], potato virus X [130], TRV [131], and several more [18] have been successfully exploited in recent years. These viruses mostly possess positive-sense RNA genomes. In addition to RNA viruses, geminiviruses have also been used for vaccine production [132].

## 9. Viroids for Generating Circular RNA

Viroids are the first group of circular RNAs identified in nature [133]. Increasing evidence revealed that circular noncoding RNAs widely exist in many organisms across the Tree of Life [134,135,136,137]. Importantly, many endogenous circular RNAs have been shown to play regulatory roles in gene expression, development, disease, etc. [134,135,138,139]. It is noteworthy that the delivery of synthetic circular RNAs has led to the suppression of gastric carcinoma cell proliferation, as a novel means of therapy [140]. It is desirable to develop a robust expression system for generating circular RNA in large quantities for functional studies and potentially for clinical therapy. Although several methods have been developed using either a cascade of enzymatic reactions [141] or the intron backsplicing mechanism [142], these systems can only reach a moderate production rate. 

As single-stranded circular noncoding RNAs, viroids can co-opt host machinery to achieve replication and systemic trafficking [43,143]. Interestingly, members in the family *Avsunviroidae* possess ribozyme activity, which is among the first groups of ribozymes identified in nature [144]. The hammerhead ribozyme in those viroids is critical to complete the infection cycle in chloroplasts [43,143,145]. Studies showed that the hammerhead ribozyme cleaves viroid RNA to generate 5′-hydroxyl and 2′,3′-phosphodiester termini, which are subsequently ligated by the chloroplast-localized tRNA ligase [146].

Based on this pathway, co-expressing the eggplant latent viroid (ELVd)-based construct and the recombinant tRNA ligase in bacteria resulted in high yield of circular ELVd RNA [147]. Interestingly, inserting exogenous sequences at a particular position of the ELVd molecule allowed the production of chimeric circular RNAs to desirable concentrations (Figure 1B) [148,149]. This circular RNA expression system provides higher production of desired RNAs in the circular form that exceeds the expression level in vivo, which will facilitate studies on circular RNA biology and its application [148,149]. 

## 10. Summary and Perspectives

In the mid-1980s, the need to purify large quantities of viroids for structural studies led to the development of a silica gel-based method [150,151]. This method was also demonstrated to be suitable for separating supercoiled plasmids from crude bacterial extracts, leading to the most popular commercial miniprep kit of Qiagen [145]. Along with the progress in nucleic acid purification techniques, structural analysis on viroid RNAs during the same period led to the recognition of suboptimal structures when certain base pairs did not belong to the deduced structure with the minimum free energy [152]. This concept constitutes a critical component in computational programs for the in silico prediction of RNA secondary structures [153], which greatly enhances the capacity and accuracy [145]. This is simply one of the many stories in history demonstrating that plant virology research has markedly contributed not only to basic sciences but also biotechnology. Recent progress in high throughput sequencing and bioinformatic tools has provided unprecedented power using small RNA sequencing to identify novel viruses and viroids from biological samples without pre-existing knowledge of viral sequences [154,155], which will uncover novel viruses to engineer suitable viral vectors for economically important crops. As plant virology research centers around the major questions in agriculture and basic sciences, it is certain that new discoveries will continue to deliver promising tools for biotechnology in the future.

## Figures and Tables

**Table 1:** Strategies to engineer VIGS vectors for major crops.

Family	Virus	Strategies to Design Vectors
*Alphaflexiviridae*	potato virus X [48]	Duplication of the subgenomic (sg) RNA promoter of the coat protein (CP) to flank a multiple cloning site between two CP sgRNA promoters
	foxtail mosaic virus [54,55]	Insertion of the *Xba*I and *Xho*I sites immediately after the stop codon of the capsid protein gene [54] Or Duplication of CP subgenomic promoter to flank a multiple cloning site [55]
*Betaflexiviridae*	citrus leaf blotch virus [56]	Inserting a subgenomic RNA promoter followed by a *Pml*I site for inserting foreign sequences in the linear form or in the hairpin fashion
	grapevine virus A [50]	Duplication of Movement Protein (MP) subgenomic RNA promoter to flank a multiple cloning site
*Bromoviridae*	cucumber mosaic virus [57]	Replacing a portion at the 3′-end of ORF2b with a multiple cloning site in RNA-2
	prunus necrotic ringspot virus [58]	Inserting foreign sequences at the 3’end of the CP gene in RNA3; Combining RNA1 and RNA2 in the same binary vector to increase the efficiency
	brome mosaic virus [59]	Using the *Hind*III site in the RNA3 3′ untranslated region (UTR) for insertion; Replacing the *Bcl*I/*Bss*HII flanked RNA3 intergenic region of the *Festuca arundinacea* strain with that from the Russian strain
*Caulimoviridae*	rice tungro bacilliform virus [60]	Selectively keeping ORFIII and a 50 bp 3′-truncated ORF IV flanked by two constitutive promoters; adding a tRNA binding site essential for replication immediately after the first promoter near the 5′-end; adding a multiple cloning site immediately before the second promoter near the 3′end
*Geminiviridae*	tomato yellow leaf curl China virus [45]	Replacing the βC1 (pathogenic factor/VSR) ORF with a multiple cloning site in DNA β
	african cassava mosaic virus [47]	Replacing a portion of the capsid protein (AV1) ORF with a multiple cloning site in DNA-A
	cotton leaf crumple virus [46]	Replacing the CP gene with a multiple cloning site in DNA-A
*Secoviridae*	broad bean wilt virus [61]	Inserting a cloning site immediately after the stop codon of the RNA2 ORF in the 3′UTR of RNA2
	bean pod mottle virus [52]	Duplication of the protease site between MP and L-CP in RNA2 to flank a multiple cloning site
	tobacco ringspot virus [53]	Duplication of the C/A protease site between MP and CP in RNA2 to flank a multiple cloning site
	apple latent spherical virus [51]	Duplication of the Q/G protease site between 42KP and Vp25 in RNA2 to flank a multiple cloning site
*Tymoviridae*	turnip yellow mosaic virus [62]	Inserting a cloning site immediately downstream the CP protein for inserting foreign sequence in the hairpin fashion; Duplicating the CP stop codon to keep the tRNA-like structure for infectivity
*Virgaviridae*	tobacco rattle virus [44]	Replacing non-structural genes in RNA2 with a multiple cloning site
	pea early browning virus [63]	Replacing non-structural genes in RNA2 with a multiple cloning site
	barley stripe mosaic virus [64]	Inserting a multiple cloning site downstream of the γb (pathogenic factor/VSR) corresponding to the γ subgenomic RNA
	cucumber green mottle mosaic virus [49]	Duplication of the CP subgenomic RNA promoter to flank a *Bam*HI site

**Table 2:** Viral VIGS vectors for major crops.

Major Crops	Viral VIGS Vectors
**Tomato**	apple latent spherical virus [51], tomato yellow leaf curl China virus [45], tobacco rattle virus [68]
**Pepper**	apple latent spherical virus [69], tobacco rattle virus [70], broad bean wilt virus2 [61]
**Potato**	tobacco rattle virus [71], potato virus X [48]
**Cassava**	african cassava mosaic virus [47,72]
**Legume**	apple latent spherical virus [51], bean pod mottle virus [52,73,74], cucumber mosaic virus [57], pea early browning virus [63], tobacco ringspot virus [53]
**Cucurbits**	apple latent spherical virus [51], tobacco ringspot virus [53], cucumber green mottle mosaic virus [49]
**Spinach**	cucumber mosaic virus [75]
**Cabbage**	turnip yellow mosaic virus [62]
**Cotton**	tobacco rattle virus [76], cotton leaf crumple virus [46]
**Citrus**	citrus leaf blotch virus [56]
**Banana**	cucumber mosaic virus [77]
**Strawberry**	tobacco rattle virus [78], apple latent spherical virus [79]
**Apple**	apple latent spherical virus [80]
**Pear**	apple latent spherical virus [80]
**Peach**	prunus necrotic ringspot virus [58]
**Grape**	grapevine virus A [50]
**Wheat**	foxtail mosaic virus [55], barley stripe mosaic virus [81]
**Barley**	foxtail mosaic virus [55], barley stripe mosaic virus [64], Brome mosaic virus [59]
**Rice**	brome mosaic virus [59], rice tungro bacilliform virus [60]
**Maize**	brome mosaic virus [59], foxtail mosaic virus [54]
**Sorghum**	brome mosaic virus [82]
**Foxtail millet**	foxtail mosaic virus [55]
**Ginger**	barley stripe mosaic virus [83]

**Table 3:** Plant virus-induced genome editing system.

	Viral Vectors	Guide RNA Design	Edited Plants	Inheritable
Dicot	cabbage leaf curl virus	U6p::gRNAScaffold::U6t inserted to the cloning site downstream of AL3	Transgenic *Nicotiana benthamiana* over-expressing Cas9 [87]	No
	tobacco rattle virus	PEBV::gRNAScaffold-Rz inserted to pTRV2 vector [85]; A FT fragment inserted at the 3′-end of gRNA resulting in a mobile sgRNA [97]	Transgenic *Nicotiana benthamiana* over-expressing Cas9 [85,97]	Yes
	bean yellow dwarf virus	Replacing MP and CP with U6::gRNAscaffold::U6t and 35S::Cas9; Agrobacterium-based transformation required for delivery	Wildtype *Nicotiana tabacum* [84]	NA
			Wildtype potato (Tetraploid and diploid) [88,89]	Via tissue culture [88]
			Wildtype tomato [90]	NA
	tobacco mosaic virus	A fragment containing the gRNAScaffold with or without a Rz inserted to the TRBO vector; 35S::Cas9 expressed from a different binary vector	*Nicotiana benthamiana* 16C [91]	NA
	potato virus X	gRNAScaffold driven by PVX CP promoter; tRNA flanking not needed	Transgenic *Nicotiana benthamiana* over-expressing Cas9 [93]	Via tissue culture
	sonchus yellow net rhabdovirus	gRNAScaffold (flanked by tRNAs) and Cas9 inserted between N and P genes under the control of duplicated N/P junction sequences	Wildtype *Nicotiana benthamiana* [92]	Yes
	beet necrotic yellow vein virus	gRNAScaffold fused to the 3′-end of the p31 ORF	Transgenic *Nicotiana benthamiana* over-expressing Cas9 [95]	NA
	foxtail mosaic virus vectors	U6p::gRNAScaffold or Cas9 inserted between duplicated CP subgenomic promoters; Mixing of gRNA and Cas9 clones for infection [86]	Transgenic *Nicotiana benthamiana* over-expressing Cas9 [104] or tomato bushy stunt virus P19 [86]	Yes if directly inoculating seeds [86]
	barley stripe mosaic virus	See below	Transgenic *Nicotiana benthamiana* over-expressing Cas9 [96]	Via tissue culture
Monocot	foxtail mosaic virus vectors	Inserting gRNAScaffold after a duplicated ORF5 promoter	Transgenic maize over-expressing Cas9 [104]	NA
			Transgenic *Setaria viridis* over-expressing Cas9 [104]	No
	wheat dwarf virus (WDV)	Replacing the MP and CP genes with Ubi::Cas9 and U6p::gRNAscaffold; T-DNA insertion procedures required	Wildtype wheat [105]	NA
		Replacing MP and CP with U6p::gRNAscaffold; Adding Ubi::Cas9::NOS in the binary vector but outside of the WDV replicon	Wildtype rice and transgenic rice over-expressing Cas9 [106]	NA
	barley stripe mosaic virus	Replacing CP with sgγ::gRNAScaffold in RNAβ or inserting gRNAScaffold immediately downstream of γb in RNAγ	Transgenic wheat over-expressing Cas9 [96]	NA
			Transgenic maize over-expressing Cas9 [96]	NA

NOTE: U6p: U6 promoter; U6t: U6 terminator; PEBV: pea early-browning virus; Rz: ribozyme; FT: flowering locus T; MP: movement protein; CP: coat protein; Ubi: ubiquitin. NA: Not Assessed.
